# Is plantar foot sensation affected in patients with gonarthrosis

**DOI:** 10.5152/j.aott.2021.21213

**Published:** 2021-11-01

**Authors:** Laçin Naz Taşcılar, Defne Kaya Utlu, Çetin Sayaca, Gökhan Polat, Ersin Kuyucu, Mehmet Emin Erdil

**Affiliations:** 1 Department of Physiotherapy, Vocational School of Health Services, Fenerbahçe University, İstanbul, Turkey; 2 Department of Physiotherapy and Rehabilitation, Faculty of Health Sciences, Bursa Uludağ University, Bursa, Turkey; 3 Department of Orthopaedics and Traumatology, İstanbul University Çapa Medical Faculty, İstanbul, Turkey; 4 Clinic of Orthopaedics and Traumatology, Medical Park Hospital, İstanbul, Turkey; 5 Department of Orthopaedics and Traumatology, Acıbadem Maslak University Hospital, Acıbadem University, İstanbul, Turkey

**Keywords:** Osteoarthritis, Plantar foot sensation, Early-stage, Late-stage, Tampa kinesiophobia, Timed Up and Go test

## Abstract

**Objective:**

The aim of this study was to compare pain, plantar foot sensation, postural control, fear of movement, and functional level between women patients with early-stage gonarthrosis and those with late-stage gonarthrosis.

**Methods:**

A total of 62 women with gonarthrosis were included in the study. Patients were then divided into two groups: early-stage gonarthrosis group (31 women) and late-stage gonarthrosis group (31 women) according to Kellgren Lawrence criteria. Light touch-pressure sensation (Semmes Weinstein Monofilaments), two-point discrimination sensation (esthesiometer), and vibration sensation (128 Hz diapason) were used to evaluate plantar foot sensation. Pain intensity was assessed by the numeric rating scale, postural control by Berg balance scale, fear of movement by the Tampa kinesiophobia scale, functional mobility by the Timed Up and Go test and knee injury and osteoarthritis outcome score.

**Results:**

Early-stage patients were found to have higher light-touch pressure sensation on 1st metatarsal head of dominant side, 5th metatarsal head of non-dominant side, heel of non-dominant side than late stage patients. Early-stage patients had a higher sensation of vibration than late stage patients. The patients in the early stage were found to have higher two-point discrimination sensation on middle of dominant side, heel of dominant side, trans-metatarsal of non-dominant side, middle of non-dominant side, heel of non-dominant side than late stage. Postural control of early-stage patients were found to be higher than late-stage patients. Early-stage patients had lower kinesophobia and higher functional levels than late-stage patients.

**Conclusion:**

The light touch sensation, vibration sensation, and two-point discrimination deteriorated by the progression of the disease should be important criteria in patients with gonarthrosis.

**Level of Evidence:**

Level III, Therapeutic Study

## Introduction

Plantar foot sensation directly stimulates the receptors under the sole, and the plantar pressure distribution is provided by the central nervous system with afferent information.^1,2^ Plantar foot senses are important for posture and gait control because only the plantar face of the foot is in contact with the ground during upright posture.^2,3^ Plantar foot afferent inputs provide information to the central nervous system for balance.^3,4^ Decreased plantar foot inputs lead to increased body oscillation and variable pressure distribution during walking.^3,5^

The main findings of osteoarthritis are joint pain, stiffness, and limitation of movement. Pain throughout the range of motion, joint stiffness, joint effusion, valgus/varus deformity, reduced physical function, lateral instability, crepitation of the patellofemoral/tibiofemoral joint with motion, and significant weakness in muscles around the knee joint (especially in the quadriceps femoris muscle) are the findings of gonarthrosis. Pain severity, postural control, walking speed, and functional level get worsened with the progression of the gonarthrosis stages.^6,7^ Additionally, patients with gonarthrosis have a loss of balance and fear of movement.^8,9^ Gait biomechanics is adversely affected in patients with knee osteoarthritis.^10^ Walking speed in patients with gonarthrosis is significantly lower than healthy individuals.^11^ Therefore, the hypothesis was that the plantar foot sensation could be impaired in individuals with gonarthrosis.

No study has investigated plantar foot sensations in the early and late stages of gonarthrosis. The aim of this study was to analyze plantar foot sensation in the early and late stages of gonarthrosis. The hypothesis of the study was that plantar foot sensation would decrease in the late stages of gonarthrosis.

## Materials and Methods

### Design

The present study was designed as a convenience sample study.

### Participants

Patients who reported to Department of Orthopedics and Traumatology with knee pain and were diagnosed to have unilateral or bilateral symptomatic gonarthrosis according to American College of Rheumatology (ACR) criteria^12^ were included in the study. Patients were divided into two groups: group I: early-stage gonarthrosis (stages 1 and 2) and group II: late-stage gonarthrosis (stages 3 and 4) according to Kellgren-Lawrence criteria.^13^ Patients’ age, height, and weight were noted.

The patients were informed on the scope and procedures of the study. All individuals provided written informed consent before participating in the study. Inclusion criteria were age ≥50 years, knee pain for at least 6 months, and diagnosis of primary gonarthrosis in radiological and clinical examination. Patients diagnosed with secondary gonarthrosis and had history of orthopedic surgery, intra-articular injection in the last 3 months, clinical or radiological meniscus, cartilage and/or ligament problem, severe physical trauma and severe psychological trauma in the last 3 months, arterial/venous circulatory disorders, skin lesion on knee joint, neuromuscular disease and/or neurological deficit, vertigo, diabetes mellitus, and auditory/vision problem were excluded from the study.^1^

### Ethics statement

This study was approved by the institutional review board of our university (Decision Number: 19). All procedures involving human participants were in accordance with the 1964 Helsinki declaration and its later amendments.

### Procedure

The symptomatic knees of the patients were evaluated. The patients performed a 5-minute bicycle warm-up before the test process. Pain intensity, plantar foot sensation, balance, fear of movement, and functional level were recorded.

#### Pain intensity:

Pain intensity was assessed using an 11-point numerical rating scale during the most painful activity (0 [no pain] to 10 [worst imaginable pain]).^14^

#### Plantar foot sensation:

**Figure 1.**
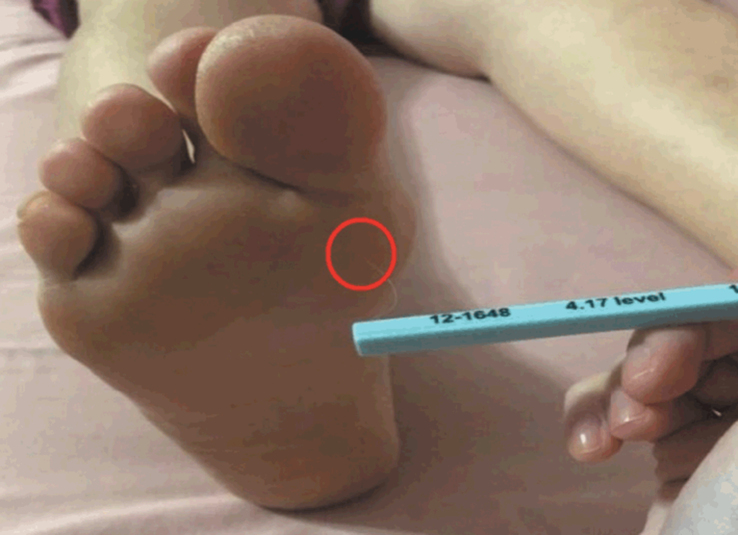
The evaluation of light-touch pressure sensation of the first metatarsal head by using Semmes-Weinstein Monofilament test kit (North Coast Medical, San Jose, CA, USA).

**Figure 2.**
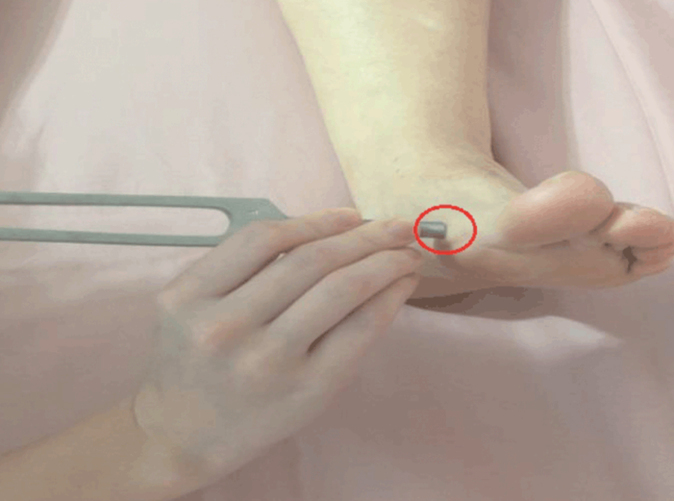
The evaluation of vibration sensation of the first metatarsal head by using 128-Hz frequency tuning fork (Elcon1 Medical Instruments,Tuttlingen,Germany).

Light-touch pressure sensation, vibration sensation, and two-point discrimination sensation were used to determine plantar foot sensation of the patients. Light-touch pressure sensation was assessed using a full Semmes-Weinstein monofilament test kit (North Coast Medical, San Jose, CA, USA) at the three regions of the foot (first metatarsal head, fifth metatarsal head, and heel) (see Figure 1). The smallest (1.65) monofilament was used first. Monofilaments were touched for 1.0–1.5 seconds to the test locations, and 1.65–3.61 monofilaments were applied three times consecutively. When the patient felt the stimulus correctly in one of the three trials, the filament representing a specific force was noted as the patient’s score (3.84), and higher filaments were applied once.^15^ Duration of vibration sensation was measured using 128-Hz frequency tuning fork (Elcon1 Medical Instruments, Tuttlingen, Germany) at the first metatarsal head, medial malleolus of the foot (Figure 2). The duration of the vibration was measured by chronometer, started when the fork touched the patient’s skin and stopped when the patient told “it has finished.” The average of the three trials was recorded in seconds.^16^ Two-point discrimination sensation of the foot sole was evaluated using an esthesiometer (Baseline1, White Plains, New York, USA) from trans-metatarsal, midpoint, and heel regions. Assessment was started at the maximum distance and gradually decreased until the patient was not able to differentiate between the two points. When the patient felt two points as one in two of the three trials, the distance was noted in millimeter.^17^

#### Balance:

Berg Balance Scale was used to evaluate functional balance performance of the patients.^18^ Turkish version of Berg Balance Scale was used.^19^

#### Fear of movement:

Tampa Kinesiophobia Scale (TKS) was used to measure the fear of movement.^20^ Turkish version of the TKS was used.^21^

#### Functional level:

Time Up and Go (TUG) test and Knee Injury and Osteoarthritis Outcome Score (KOOS) were used to determine the functional level of the patients. The patients were asked to return, sit back in the chair after getting up, and walk 3 m away.

The test was repeated three times, and the average of the duration was recorded.^22^ KOOS includes five subscales: pain, symptoms, activities of daily living, sport and recreational function (Sport/Rec), and knee-related quality of life.^23^ The Turkish version of KOOS was used.^24^

### Statistical analysis

All data were analyzed with the Statistical Package for the Social Sciences (SPSS) (IBM Corp., Armonk, NY, USA) version 22. Characteristics of patients were described using means and standard deviations. The normality of the distribution of the data was investigated by Shapiro–Wilk testing with α set at 0.05. Descriptive statistics (mean, standard deviation, frequency) were used for the evaluation of study data, and Student’s *t*-test was used for the normal distribution of the quantitative data between the two groups. Mann–Whitney *U*-test was used for those who did not show normal distribution. Kruskal–Wallis test was used for multiple group comparisons, and Spearman correlation test was used to investigate the relationship between variables. Statistical significance was set at α ≤ 0.05.

## Results

A total of 31 women with stages 1 and 2 (early) gonarthrosis and another 31 women with stages 3 and 4 (late) gonarthrosis (total 121 knees, [59 bilateral, 3 unilateral]) completed assessment procedures in the present study. The sample size was determined using G*Power 3.1.9.2 program. Alpha: 0.05, effect size: 0.8, group I: 31 and group II: 31; a total of 62 cases were included in the study. The age, height, and body mass index (BMI) of the patients are presented in Table 1.

### Comparison variables of the patients between early and late stages

#### Pain:

There was no statistically significant difference while the pain intensity of the patients in the early stage was lower than in the late stage (*P* > 0.05) (Table 2).

#### Postural control:

The postural control of the patients in the early stage was higher than that of the patients in the late stage (*P* ≤ 0.05) (Table 2).

#### Fear of movement:

Fear of movement level of the patient in the early stage was lower than that of the patients in the late stage (*P* ≤ 0.05) (Table 2).

#### Functional level:

TUG completion time of the patient in the early stage was lower than that in the late stage (*P* ≤ 0.05) (Table 2). KOOS of the patients in the early stage was higher than that of the patients in the late stage (*P* ≤ 0.05) (Table 2).

#### Plantar foot sensation:

In the early stage, the light-touch pressure sensation of the first metatarsal head, fifth metatarsal head, and midpoint of the heel was found to be higher than that in the late stage (*P* ≤ 0.05) (Table 3). Vibration sensation of the patients in the early stage was higher than that of the patients in the late stage (*P* ≤ 0.05) (Table 4). Patients in the early stage had higher trans-metatarsal, sole midpoint, and heel two-point discrimination sensation than those in the late stage (*P* ≤ 0.05) (Table 5).

### Relation between plantar foot sensation and other variables

#### Light touch-pressure sensation:

The relations of light-touch pressure sensation with pain, postural control, fear of movement, and functional level are shown in Table 6. A positive correlation was found between pain severity and light-touch pressure monofilament value of the right heel, left first metatarsal tip, fifth metatarsal tip of the patients in the early stage. A negative correlation was found between postural control and light-touch pressure monofilament value of the right heel, left first metatarsal tip, fifth metatarsal tip in the early stage. There was a positive correlation between fear of movement and light-touch pressure monofilament value of the right foot (all regions), left fifth metatarsal tip of the patients in the early stage. A positive correlation was found between completion duration of TUG test and light-touch pressure monofilament value of the right heel, left fifth metatarsal tip of the patients in the early stage, right fifth metatarsal tip, left first metatarsal tip, and fifth metatarsal tip of the patients in the late stage. A negative correlation was found between KOOS and light-touch pressure monofilament value of right–left foot (all regions) of the patients in the early stage.

#### Vibration sensation:

The relations between vibration sensation and pain, postural control, fear of movement, and functional level are shown in Table 6. A negative correlation was observed between pain intensity and vibration sensation of left first metatarsal tip of the patients in the early stage. There was a positive correlation between postural control and vibration sensation of right first metatarsal tip and left first metatarsal tip of the patients in the early stage. A negative correlation was found between fear of movement and vibration sensation of right–left foot (all regions) of the patients in the early stage. There was a negative correlation between the completion duration of TUG and vibration sensation of right–left first metatarsal tip of the patients in the early stage. A positive correlation was found between KOOS and vibration sensation of right–left first metatarsal tip of the patients in early stage.

#### Two-point discrimination sensation:

The relationships of two-point discrimination sensation with pain, postural control, fear of movement, and functional level are shown in Table 6. There was a positive correlation between the pain severity and two-point discrimination of right trans-metatarsal, sole midpoint and left sole midpoint, and heel midpoint of the patients in the early stage. There was a negative correlation between postural control and two-point discrimination of right foot (all regions), left trans-metatarsal, heel midpoint of the patients in early stage, and right–left heel midpoint of the patients in the late stage. A positive correlation was observed between fear of movement and two-point discrimination of the right sole midpoint of the patients in the early stage and right heel midpoint of the patients in the late stage. There was a positive correlation between the completion duration of TUG test and two-point discrimination of right trans-metatarsal, sole midpoint, left trans-metatarsal, heel midpoint of the patients in the early stage and right sole midpoint, heel midpoint, left trans-metatarsal, heel midpoint of the patients in the late stage. There was a negative correlation between KOOS and two-point discrimination of right trans-metatarsal, sole midpoint, left sole midpoint, heel midpoint of the patients in the early stage.

## Discussion

While planning this study, we asked ourselves if there was any change in the protective plantar foot sensation in patients with gonarthrosis. Both the protective plantar foot sensation and gonarthrosis are negatively affected with age. This is the first study to examine protective plantar foot sensation in gonarthrosis.

The principal findings of the study were plantar foot sensation (light touch-pressure, vibration, two-point discrimination) of the patients worsened with the progression of the disease to the late stage. The plantar foot sensation was negatively affected with the progression of the radiological stage in patients with gonarthrosis. It is known that plantar foot sensation is related to balance and postural control in patients with type 2 diabetes mellitus, multiple sclerosis, and hemodialysis.^1,4,25^ It was the first study that investigated plantar foot sensation and compared between early and late stage gonarthrosis in the literature. Decreased light-touch pressure sensation was found in the early stage. Patients’ light-touch pressure sensations were found to be more impaired with the progression of the disease to the late stage radiologically. These results made us believe that light-touch pressure sensation is an important finding in the gonarthrosis process. Vibration sensation of the patients was found to be worsened with the progression of the disease to the late stage radiologically. The present study reported that vibration sensation was one of the main findings in patients with gonarthrosis and should be examined in the future studies. When we examined the sense of two-point discrimination, we found that in the early-stage gonarthrosis, the difference of the two-point discrimination was not impaired, and the sense of two-point separation in the late phase was decreased.

### Plantar Foot Sensation and Relations with Other Parameters

#### Plantar foot sensation and pain:

Rosenbaum et al. reported that there was no relationship between pain during walking and light-touch sensation in patients with rheumatoid arthritis.^26^ There is no study that investigated the relationship between pain severity and plantar foot sensation. The present study is the first one to show that plantar sensation is related to pain severity in patients with gonarthrosis. More studies on the relationship between plantar foot sensation and pain severity in patients with gonarthrosis are needed because pain is one of the main symptoms of gonarthrosis.

#### Plantar foot sensation and postural control:

Decreased plantar foot sensation caused impairment of individuals’ balance and increased risk of falling.^5,27^ Kafa et al. found that patients’ plantar foot sensations increased with increasing balance of patients with type 2 diabetes. They also found that only the light-touch pressure sensation was associated with balance, which is one of the plantar foot sensation evaluation variables.^4^ Wang et al. found that patients with decreased plantar foot sensation had more postural sway.^28^ This study reported that worsened postural control of the patients with gonarthrosis in the early stage was related with decreased light touch sensation. Thus, we believe plantar foot sensation should be examined in the evaluation of postural control in patients with gonarthrosis in future studies. Citaker et al. found that the vibration sensation of the first metatarsal head increased with the increase in the duration of one leg stance in patients with multiple sclerosis.^1^ In our study, it was found that worsened postural control of the patients with gonarthrosis in the early stage related with decreased vibration sense. This similarity between two studies may be attributed to that big toe carries the highest load while standing. Menz et al. evaluated the plantar foot sensation of the healthy individuals by using an esthesiometer. They found that individuals with lower plantar foot sensation fell more. People with decreased plantar foot sensation are reported to fall more than those with normal plantar foot sensation.^29^ The present study showed that worsened postural control related with decreased two-point discrimination. We believe plantar foot sensation is an indicator of damaged postural control. Thus, the evaluation of plantar foot sensation should be included into the treatment of gonarthrosis in future studies.

#### Plantar foot sensation and fear of movement:

Eils et al. found that patients with decreased plantar foot sensation move more carefully in push-off phase during walking.^3^ In our study, we found that increased fear of movement related with decreased plantar foot sensation in patients with gonarthrosis. Hafström also found that patients with decreased plantar foot sensation had low walking speed.^30^ These results showed a relationship between fear of movement and plantar foot sensation. The present study showed that fear of movement was related to plantar foot sensation in patients with gonarthrosis.

#### Plantar foot sensation and functional level:

Hafström found that the plantar foot sensation was very important in functional balance.^30^ Our study showed that prolonged time of TUG test of the patients with the early-stage and late-stage gonarthrosis related with decreased plantar foot sensation. It was also shown that decreased functional level, which was evaluated subjectively using KOOS, was related to decreased plantar foot sensation in patients with the early-stage gonarthrosis. Thus, the plantar foot sensation assessment is believed to be one of the crucial assessments in gonarthrosis, especially for the early stage. We believe it is needed to examine the plantar foot sensation in patients with gonarthrosis to control the radiological process of gonarthrosis because of the relationship between plantar foot sensation and radiological stage of gonarthrosis.

Plantar foot sensation decreases with increasing age.^30^ The incidence of gonarthrosis increases with age.^31^ With aging, the decrease in postural control and functional level becomes an important problem in patients with gonarthrosis.^32,33^ This study investigated the relationship between plantar foot sensation loss and degenerative changes. Studies with long follow-up periods starting from the early age and early stage of gonarthrosis are required.

In the present study, it was determined that three criteria (light touch, vibration, and two-point discrimination) worsened with the progression of the disease to the late stage. Decreased plantar foot sensation is shown as one of the symptoms of impaired postural control in individuals.^30,34^ Therefore, we attribute the impairment of plantar foot sensation with the radiological progression of the disease to the fact that the postural control of the patients was gradually affected negatively and therefore the patients became recessive from the activity. There was no study on the responsible pathways for impaired plantar foot sensation in patients with gonarthrosis. Similar to the relationship between falling and decreased plantar foot sensation that develops with advancing age, we think that the treatments for plantar foot sensation in the early stages of gonarthrosis will prevent the balance of patients from getting worse during the disease process. Studies investigating the neural pathways of the relationship between different stages of gonarthrosis and decreased plantar foot sensation are needed.

This study has several limitations. The distributions of 62 individuals were as below: stage I, 2; stage II, 29 people in group I; stage III, 19; stage IV, 12 people in group II. This inequal distribution in stages is a limitation. Studies that consist of an equal number of patients for all stages are needed.

BMI of the patients included in the study was high. An evaluation that examines the effect of subcutaneous adipose tissue thickness on plantar foot sensation and postural control was not included in the study. Studies evaluating subcutaneous tissue thickness with ultrasound imaging method or skinfold caliper are needed.

No rehabilitation approach was applied in this study for patients with gonarthrosis. Studies investigating the effect of rehabilitation approaches to improve plantar foot sensation, postural control, and performance on gonarthrosis symptom, severity, and process are needed.

The light touch, vibration, and two-point discrimination values of the plantar foot sensation deteriorated/affected as the disease progressed to the late stage. This result indicated that plantar foot sensation may be an indicator with the progression of the disease to the late stage. Exercise and/or rehabilitation methods to improve these three sensations that are impaired/affected at an early stage can slow down the osteoarthritis process. There is a need for studies related to this.

Light-touch sensation that was found to be adversely affected by gonarthrosis progression was also found to be decreased in patients with early-stage gonarthrosis. Light-touch sensation, which was found to be impaired in the early stage, was an important finding in the treatment of gonarthrosis, and treatment modalities should be included in the treatment program in order to prevent the progress of the disease.

## The literature review of plantar foot sensation

Plantar foot sensation is very important in terms of posture and gait control because only the plantar aspect of the foot is in contact with the ground during upright posture.^1-^^3^ Afferent inputs of the plantar foot provide information to the central nervous system for the formation of balance. When these inputs are not transmitted to the central nervous system, balance disorder may develop.^1^

Decreased plantar foot input causes increased body sways while standing and altered pressure distribution during walking. Decreased plantar foot sensation causes significant changes in gait pattern, and decreased light touch sensation has been shown to be highly associated with falling in the elderly.^3^

Plantar foot receptors play an important role in maintaining standing balance. Bilateral loss of somatosensory information in the foot causes increased body sway, resulting in postural instability.^4^ The light-touch sensation has as much effect as the activity of the tibialis anterior muscle on the lower extremity joints in controlling the gait pattern.^2^

Individuals with gonarthrosis have impaired balance control compared to healthy individuals, and dynamic balance is more affected than static balance.^35,36^ In addition, patients with gonarthrosis have an increased risk of falling.^37^

The decreased plantar foot sensation causes the balance disorder and increased risk of falling.^5,28^ Kafa et al. found that the light-touch sensation of the patients increased with the increase in balance, which they evaluated by measuring the time of standing on one foot in patients with type 2 diabetes. They also found that only light touch sensation, which is one of the evaluation variables for the plantar foot sensation, was associated with balance.^4^ Wang et al. found that patients with decreased plantar foot sensation had more postural sway.^28^ Patients with gonarthrosis suffer from balance disorder, and balance is associated with the plantar foot sensation, thus we believe that plantar foot sensation decreases in patients with gonarthrosis.

This study was initiated to see if the protective plantar foot sensation was affected in gonarthrosis and was completed with many questions such as “What is the neural mechanism of this decreased protective plantar foot sensation we found?,” “Is this loss of protective plantar foot sensation that we determined in gonarthrosis caused by age or due to the long-term negative loading on the sole of the foot because of biomechanical disorders as a result of gonarthrosis?,” “Does plantar foot sensation training work in patients with early-stage gonarthrosis progressing to late stages?”

**Highlights**

- Plantar foot sensation, postural control, and performance are adversely affected in the early stages of gonarthrosis.
- Plantar foot sensation, postural control, and performance may have a role in reducing the symptoms and severity of gonarthrosis.
- The effect of rehabilitation programs that include approaches to plantar foot sensation on symptoms and process of gonarthrosis should be evaluated.
- Rehabilitation approaches that improve plantar foot sensation, postural control, and performance should be applied to the early-stage patients with gonarthrosis in future studies and clinical applications.

## Figures and Tables

**Table 1.** Demographic Information

Demographic Information	Early Stage		Early Stage (n = 31)	Late Stage		Late Stage (n = 31)	Early Stage − Late Stage
	Stage 1 (n = 2)	Stage 2 (n = 29)		Stage 3 (n = 19)	Stage 4 (n = 12)		
	X ± SD	X ± SD	X ± SD	X ± SD	X ± SD	X ± SD	*P* *
Age	55.5 ± 6.36	58.52 ± 6.83	58.32 ± 6.75	63.58 ± 8	66.75 ± 9.16	64.81 ± 8.46	0.00*
Height (cm)	165.5 ± 3.53	158.5 ± 5.17	159.03 ± 5.33	158.2 ± 4.4	155 ± 2.9	157.03 ± 4.15	0.11
Weight (kg)	67.5 ± 3.53	76.21 ± 12.55	75.65 ± 12.34	80.4 ± 15.04	81.75 ± 12.71	80.94 ± 13.98	0.12
BMI (kg/m^2^)	24.6 ± 2.34	30.3 ± 5.02	29.9 ± 5.07	32 ± 5.50	33.9 ± 4.83	32.7 ± 5.26	0.03*

n, number of participants; X, mean; SD, standard deviation; BMI, body mass index.

* Independent sample *t* test, *P* ≤ 0.05.

**Table 2.** Comparison of Pain, Postural Control, Fear of Movement, Functional Level Between the Stages of Gonarthrosis

	Early Stage		Early Stage (n = 31)	Late Stage		Late Stage (n = 31)	*z*µ	*P*µ	*P* *
	Stage 1 (n = 2)	Stage 2 (n = 29)		Stage 3 (n = 19)	Stage 4 (n = 12)				
	X ± SD	X ± SD	X ± SD	X ± SD	X ± SD	X ± SD			
Pain severity	7.00 ± 1.41	7.00 ± 1.87	7.00 ± 1.82	7.68 ± 1.73	7.58 ± 1.78	7.65 ± 1.72	−1.54	0.12	0.04*
BBS	52.00 ± 4.24	46.41 ± 11.67	46.77 ± 11.38	39.26 ± 12.37	39.08 ± 12.63	39.19 ± 12.26	−3.34	0.00*	0.00*
KP	43.00 ± 19.79	44.00 ± 9.72	43.94 ± 10.07	48.89 ± 9.01	51.83 ± 7.89	50.03 ± 8.58	−2.32	0.02*	0.09
TUG	8.79 ± 1.41	12.61 ± 9.45	12.36 ± 9.19	13.71 ± 4.88	15.60 ± 4.87	14.44 ± 4.89	−3.54	0.00*	0.00*
KOOS	49.10 ± 19.79	42.78 ± 21.21	43.19 ± 20.87	35.26 ± 19.29	23.97 ± 11.46	30.89 ± 17.40	−2.43	0.01*	0.02*

n, number of participants; X, mean; SD, standard deviation; µ, Mann–Whitney *U*-Test;

*P*^µ^, comparison of early and late stage; P^*^, comparison of stages with each other;

BBS, Berg Balance Scale; KP, Kinesiophobia; TUG, time up and go test; KOOS, Knee Injury and Osteoarthritis Outcome Score.

* Kruskal Wallis Test.

**Table 3.** Comparison of Light Touch Sensation Between the Stages of Gonarthrosis

		Early Stage		Early Stage (n = 31)	Late Stage		Late Stage (n = 31)	*z*µ	*P*µ	*P* *
		Stage 1 (n = 2)	Stage 2 (n = 29)		Stage 3 (n = 19)	Stage 4 (n = 12)				
		X ± SD	X ± SD	X ± SD	X ± SD	X ± SD	X ± SD			
Light Touch	1MT	3,63 ± 0.72	3.65 ± 0.53	3.65 ± 0.53	3.85 ± 0.50	4.19 ± 0.43	3.98 ± 0.50	−3.15	0.00*	0.00*
	5MT	3.51 ± 0.60	3.79 ± 0.49	3.77 ± 0.50	3.95 ± 0.46	4.25 ± 0.36	4.07 ± 0.44	−3.53	0.00*	0.00*
	HMP	3.69 ± 0.61	4.07 ± 0.55	4.04 ± 0.56	4.25 ± 0.38	4.48 ± 0.43	4.34 ± 0.41	−3.09	0.00*	0.00*

n, number of participants; X, mean; SD, standart deviation;

1MT, 1 metatarsal tip; 5MT, 5 metatarsal tip; HMP, heel mid-point;

µ, Mann–Whitney *U*-Test; *P*^µ^, comparison of early and late stage; P^*^: comparison of stages with each other.

* Kruskal Wallis Test.

**Table 4.** Comparison of Vibration Sensation Between the Stages of Gonarthrosis

		Early Stage		Early Stage (n = 31)	Late Stage		Late Stage (n = 31)	*z*µ	*P*µ	*P* *
		Stage 1 (n = 2)	Stage 2 (n = 29)		Stage 3 (n = 19)	Stage 4 (n = 12)				
		X ± SD	X ± SD	X ± SD	X ± SD	X ± SD	X ± SD			
Vibration	1MT	10.89 ± 1.48	9.77 ± 2.41	9.84 ± 2.37	7.99 ± 1.44	7.93 ± 2.08	7.97 ± 1.70	−4.78	0.00*	0.00*
	MM	10.43 ± 0.89	8.89 ± 1.97	8.99 ± 1.95	7.01 ± 1.54	7.98 ± 2.07	7.38 ± 1.81	−4.43	0.00*	0.00*

n, number of participants; X, mean; SD, standard deviation;

1MT, 1 metatarsal tip; MM, Medial Malleol;

µ, Mann–Whitney *U*-Test;

*P*^µ^, comparison of early and late stage;

P^*^, comparison of stages with each other.

* Kruskal Wallis Test;

**Table 5.** Comparison of Two Point Discrimination Sensation Between the Stages of Gonarthrosis

		Early Stage		Early Stage (n = 31)	Late Stage		Late Stage (n = 31)	*z*µ	*P*µ	*P* *
		Stage 1 (n = 2)	Stage 2 (n = 29)		Stage 3 (n = 19)	Stage 4 (n = 12)				
		X ± SD	X ± SD	X ± SD	X ± SD	X ± SD	X ± SD			
Two point discrimination	TM	1.82 ± 0.92	2.20 ± 1.21	2.17 ± 1.19	2.64 ± 1.59	3.89 ± 1.86	3.13 ± 1.84	−3.23	0.00*	0.00*
	SMP	2.15 ± 1.04	2.35 ± 1.50	2.34 ± 1.47	2.90 ± 1.55	3.96 ± 2.31	3.31 ± 1.93	−3.90	0.00*	0.00*
	HMP	1.60 ± 0.51	2.20 ±.1.22	2.16 ± 1.20	2.58 ± 1.08	3.33 ± 1.12	2.87 ± 1.15	−3.80	0.00*	0.00*

n, number of participants; X, mean; SD, standard deviation;

TM, trans-metatarsal; SMP, sole mid-point; HMP, heel mid-point;

µ, Mann–Whitney *U*-Test;

*P*^µ^, comparison of early and late stage;

P^*^, comparison of stages with each other.

* Kruskal Wallis Test.

**Table 6.** Relations of Plantar Foot Sensation (Light Touch, Vibration and Two Point Discrimination) with Pain, Postural Control, Fear of Movement, Functional Level in Early Stage and Late Stage of Gonarthrosis

		Light-Touch Pressure Sensation											
		Right						Left					
		1MT		5MT		HMP		1MT		5MT		HMP	
		r	*P* ^¥^	r	*P* ^¥^	r	*P* ^¥^	r	*P* ^¥^	r	*P* ^¥^	r	*P* ^¥^
Pain	a	0.263	0.15	0.256	0.16	0.423*	0.01*	0.37*	0.03*	0.40*	0.02*	0.321	0.07
	b	−0.042	0.82	−0.107	0.56	−0.108	0.56	−0.010	0.95	0.106	0.57	0.144	0.43
BBS	a	−0.322	0.07	−0.324	0.07	−0.589	0.00*	−0.467	0.00*	−0.537	0.00*	−0.353	0.51
	b	−0.119	0.52	−0.143	0.44	−0.042	0.82	−0.191	0.30	−0.158	0.39	−0.024	0.89
KP	a	0.509	0.00*	0.39*	0.03*	0.551	0.00*	0.293	0.11	0.398*	0.02*	0.280	0.12
	b	0.214	0.24	0.143	0.44	−0.208	0.26	0.167	0.37	0.172	0.35	0.122	0.51
TUG	a	0.185	0.32	0.216	0.24	0.44*	0.01*	0.261	0.15	0.43*	0.01*	0.240	0.19
	b	0.243	0.18	0.360*	0.04*	0.039	0.83	0.367*	0.04*	0.393	0.03*	0.088	0.64
KOOS	a	−0.516	0.00*	−0.480	0.00*	−0.583	0.00*	−0.44*	0.01*	−0.575	0.00*	−0.44*	0.01*
	b	−0.105	0.57	−0.173	0.35	0.066	0.72	−0.148	0.42	−0.259	0.16	−0.316	0.08

		Vibration Sensation							
		Right				Left			
		1MT		MM		1MT		MM	
		r	*P* ^¥^	r	*P* ^¥^	r	*P* ^¥^	r	*P* ^¥^
Pain	a	−0.339	0.06	−0.198	0.28	−0.446*	0.01*	−0.224	0.22
	b	0.250	0.17	0.210	0.25	0.175	0.34	0.029	0.87
BBS	a	0.494	0.00*	0.337	0.06	0.480	0.00*	0.241	0.19
	b	0.002	0.99	−0.263	0.15	0.090	0.63	−0.118	0.52
KP	a	−0.466	0.00*	−0.42*	0.02*	−0.602	0.00*	−0.36*	0.04*
	b	−0.163	0.38	0.157	0.39	−0.142	0.44	0.049	0.79
TUG	a	−0.40*	0.02*	−0.318	0.08	−0.456	0.01*	−0.304	0.09
	b	−0.056	0.76	0.075	0.69	−0.149	0.42	−0.065	0.73
KOOS	a	0.476	0.00*	0.313	0.08	0.571	0.00*	0.217	0.24
	b	−0.042	0.82	−0.40*	0.02*	0.019	0.92	−0.104	0.57

		Two Point Discrimination											
		Right						Left					
		TM		SMP		HMP		TM		SMP		HMP	
		r	*P* ^¥^	r	*P* ^¥^	r	*P* ^¥^	r	*P* ^¥^	r	*P* ^¥^	r	*P* ^¥^
Pain	a	0.42*	0.01*	0.472	0.00*	0.180	0.33	0.334	0.06	0.38*	0.03*	0.460	0.01*
	b	0.097	0.60	−0.071	0.70	0.144	0.44	−0.081	0.66	−0.040	0.83	0.182	0.32
BBS	a	−0.43*	0.01*	−0.482	0.00*	−0.410	0.02*	−0.42*	0.01*	−0.312	0.08	−0.553	0.00*
	b	−0.077	0.68	−0.349	0.05	−0.36*	0.04*	−0.237	0.20	−0.222	0.23	−0.41*	0.02*
KP	a	0.245	0.18	0.359*	0.04*	0.261	0.15	0.142	0.44	0.242	0.19	0.39*	0.03*
	b	0.137	0.46	0.202	0.27	0.308	0.01*	0.259	0.16	0.177	0.34	0.286	0.12
TUG	a	0.471	0.00*	0.38*	0.03*	0.331	0.07	0.40*	0.02*	0.296	0.10	0.487	0.00*
	b	0.281	0.12	0.534	0.00*	0.615	0.00*	0.458	0.01*	0.289	0.11	0.514	0.00*
KOOS	a	−0.39*	0.02*	−0.521	0.00	−0.289	0.11	−0.349	0.05	−0.38*	0.03*	−0.501	0.00*
	b	−0.318	0.08	−0.186	0.31	−0.37*	0.03*	−0.314	0.08	−0.146	0.43	−0.338	0.06

BBS, Berg Balance Scale; KP, Kinesiophobia; TUG, time up and go test; KOOS, Knee Injury and Osteoarthritis Outcome Score;

1MT, 1 metatarsal tip; 5MT, 5 metatarsal tip; HMP, heel mid-point; MM, medial malleol; TM, trans-metatarsal; SMP, sole mid-point; HMP, heel mid-point;

a, early stage; b, late stage; ¥, Spearman correlation analysis; r, correlation coefficient.

* *P* ≤ 0.05.
